# Morphology-Engineered CeO_2_ as a Synergistic Flame Retardant in Polypropylene/Intumescent Systems: Mechanisms and Performance Enhancement

**DOI:** 10.3390/molecules30102102

**Published:** 2025-05-09

**Authors:** Bangmin Li, Wayne Hsu, Tingyi Zheng, Yincai Wu, Shenglong Wang, Fenglong Lin, Lijun Song, Xianfa Rao

**Affiliations:** 1 JiangXi University of Science and Technology, Ganzhou 341000, Chinaraoxianfa@126.com (X.R.); 2 Xiamen Institute of Rare Earth Materials, Chinese Academy of Sciences, Xiamen 361021, China; wayne@fjirsm.ac.cn (W.H.); xmzhengtingyi@fjirsm.ac.cn (T.Z.); xmwuyincai@fjirsm.ac.cn (Y.W.); xmwangshenglong@fjirsm.ac.an (S.W.); 3 Key Laboratory of Design and Assembly of Functional Nanostructures, Fujian Provincial Key Laboratory of Nanomaterials, Fujian Institute of Research on the Structure of Matter, Chinese Academy of Sciences, Fuzhou 350002, China; 4 Xiamen Key Laboratory of Rare Earth Photoelectric Functional Materials, Xiamen 361021, China; 5 Fujian College, University of Chinese Academy of Sciences, Fuzhou 350002, China

**Keywords:** cerium oxide, morphology, intumescent flame retardant, polypropylene, synergistic effect

## Abstract

This study systematically examines the effect of the morphology of cerium oxide (CeO_2_) on the flame retardancy, thermal stability, and mechanical properties of polypropylene composites with intumescent flame retardant (PP/IFR). Layer-CeO_2_ (L-CeO_2_) outperforms Particulate-CeO_2_ (P-CeO_2_) in enhancing the flame retardancy of PP/IFR composites, showing higher limiting oxygen index (LOI) and greater reductions in the total heat release rate (THR) and total smoke production (TSR). The substitution of 1% IFR with 1% L-CeO_2_ significantly increased the LOI from 29.4% to 32.6%, while reducing the THR and TSR by 38.9% and 74.3%, respectively. L-CeO_2_ incorporation improves thermal stability, increasing the residual char yield to 8.53% at 800 °C under air (vs. 3.87% for PP/IFR). Additionally, L-CeO_2_ improved the mechanical properties of the composites, increasing tensile strength and rigidity. The synergistic flame-retardant mechanism is hypothesized to involve CeO_2_ catalyzing the formation of a P-O-C crosslinked network in the carbon layer, leading to a denser carbon structure and improved flame-retardant performance in the PP/IFR composites. These findings demonstrate the efficacy of L-CeO_2_ as a flame-retardant synergist, providing a foundation for developing fire-safe polymeric materials.

## 1. Introduction

Polypropylene (PP) is widely employed in industries such as electronics, automotive, and construction due to its low density, corrosion resistance, thermal stability, and being colorless and odorless [1,2]. However, the inherent flammability of PP (LOI = 17.8%) restricts its application in fields demanding stringent fire safety standards [1]. As a result, enhancing the flame retardancy of PP has become a critical focus of research. Intumescent flame retardants (IFRs) offer promising solutions due to their advantages of low smoke emission, non-toxicity, and anti-drip properties [3]. Typically, IFR systems consist of three essential components: an acid source, a carbon source, and a gas source [4,5,6]. Piperazine pyrophosphate (PAPP) is an effective flame retardant that satisfies these criteria, and its molecular structure, featuring a piperazine ring, endows it with a high initial decomposition temperature and excellent charring ability. Consequently, PAPP has been incorporated into various flame-retardant composites [7,8]. Melamine polyphosphate (MPP) is a melamine derivative, which is a commonly used blowing agent in IFR [7]. It can play a good synergistic role with PAPP. As an IFR, the flame-retardant effect of PAPP/MPP is mainly realized through the interaction of acid source, gas source, and carbonization source to form a continuous expanded carbonization layer on the surface to isolate oxygen and heat [9]. However, there are drawbacks, such as large additive quantity and low flame-retardant efficiency [10].

The development of synergistic flame retardants incorporating nanoclays [11,12], transition metal oxides [13,14], or rare earth compounds [15,16], has been shown to effectively enhance the flame-retardant efficiency of conventional IFR systems. The commonly used ones are alumina, zinc oxide, aluminum hydroxide, etc. [17,18]. However, the catalytic carbon formation effect of transition metal oxides is average, and metal hydroxides require a large amount of additive and are prone to causing a decrease in the mechanical properties of the materials [19]. Studies have demonstrated that rare earth oxides exhibit remarkable synergistic effects as flame retardants, acting as catalysts for dehydrogenation and esterification reactions that promote the formation of dense, porous char layers [16]. For example, Feng et al. [20] investigated the role of CeO_2_ in a PP/IFR system, where CeO_2_ catalyzed reactions between polyphosphoric acid, carbon-forming agents, and polycyclic aromatic hydrocarbons, resulting in complex crosslinked networks and improved char strength, which enhanced flame retardancy. Similarly, Ren et al. [21] studied the effects of Nd_2_O_3_ and La_2_O_3_ on a polypropylene/poly(octylene-co-ethylene) blend system with IFRs, demonstrating that rare earth oxides promote esterification and carbonization processes, thereby improving the thermal stability of the composites. However, studies on the effects of different morphologies of rare earth oxides combined with IFR on the flame retardancy of polymeric materials are relatively limited.

In this work, PAPP and MPP acted as an IFR, and CeO_2_ with distinct morphologies was incorporated as a synergistic flame retardant. The effect of CeO_2_ morphology (layered vs. particulate) on the flame retardancy, thermal stability, and mechanical properties of PP/IFR composites was systematically investigated.

## 2. Results and Discussion

### 2.1. Characterization of CeO_2_

**Figure 1:**
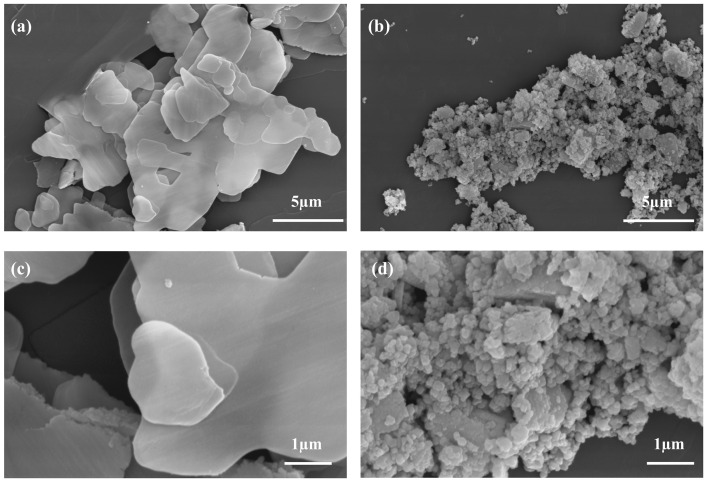
SEM images of different morphologies of CeO_2_: (**a**,**c**) Layered-CeO_2_, (**b**,**d**) Particulate-CeO_2_.

Scanning electron microscopy (SEM) images (Figure 1) revealed distinct morphologies for L-CeO_2_ and P-CeO_2_. L-CeO_2_ exhibited a flake-like structure, whereas P-CeO_2_ appeared as irregular particles. As shown in Figure S1, the diffraction peaks of L-CeO_2_ and P-CeO_2_ are consistent with the typical fluorite structure of CeO_2_ (PDF#43-1002). Meanwhile, the intensities difference between L-CeO_2_ and P-CeO_2_ corresponded to the growth orientation; for example, the (200) and (400) peaks indicated that L-CeO_2_ expanded along the two-dimensional direction, although the weaker peaks at (111), (220), and (311) may be due to the slow growth in other crystal planes [22].

The higher the absolute value of the zeta potential, the better the dispersion and stability of the particles [23]. From Table S1, it can be seen that the absolute value of the zeta potential of L-CeO_2_ is 36.78, while the absolute value of the zeta potential of P-CeO_2_ is only 18.11.

**Figure 2:**
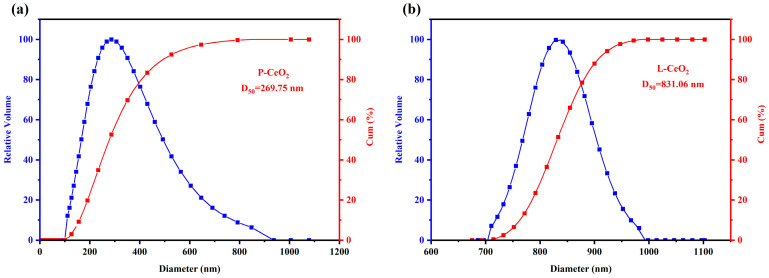
Particle size lognormal distribution model and distribution cumulative plot: (**a**) P-CeO_2_, (**b**) L-CeO_2_.

The particle size distribution of CeO_2_ with different morphologies was tested using the DLS method, and the results (Figure 2) showed that the D_50_ of P-CeO_2_ was 269.75 nm, while the D_50_ of L-CeO_2_ was 831.06 nm. Generally, nano-sized flame retardants have better flame retardancy than micron-sized flame retardants [24]. However, compared to L-CeO_2_, P-CeO_2_ particles are poorly dispersed and easily agglomerated (as shown in the zeta potential and SEM tests), which affects the flame-retardant effect.

**Figure 3:**
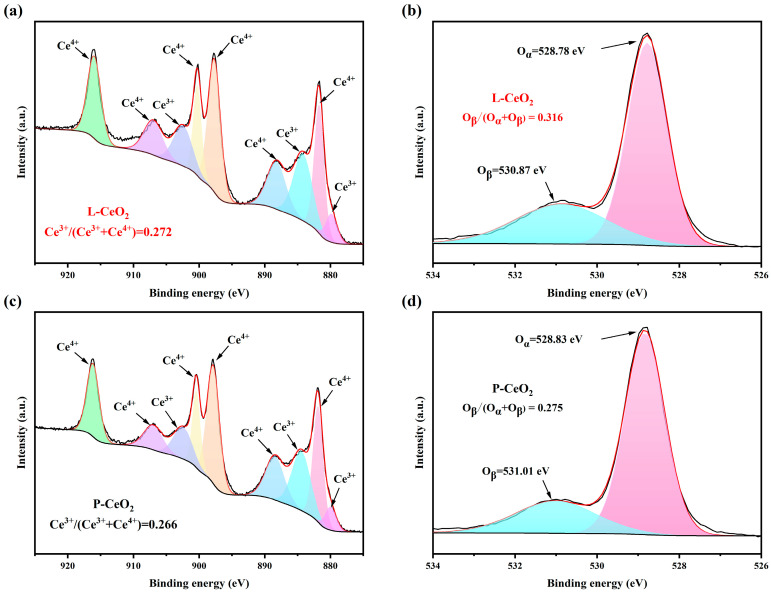
(**a**,**c**) Ce 3d XPS spectra of L-CeO_2_ and P-CeO_2_; (**b**,**d**) O 1s XPS spectra of L-CeO_2_ and P-CeO_2_.

Oxygen vacancies commonly serve as reaction sites and influence catalytic activity in many oxidation reactions; the more oxygen vacancies there are, the more efficient active sites are provided [25,26]. The ratio of Ce^3+^/(Ce^3+^ + Ce^4+^), which is the percentage content of Ce^3+^, can be calculated by fitting the peak area to the Ce 3d spectra. The formation of oxygen vacancies in CeO_2_ is usually accompanied by an increase in the content of Ce^3+^, so the larger the ratio is, the greater the content of oxygen vacancies in CeO_2_. Figure 3 shows six peaks (881.72, 897.70, 900.26, 888.23, 906.97, and 916.97 eV) for Ce^4+^ 3d and three peaks (879.82, 902.47, and 884.21 eV) for Ce^3+^ 3d [27,28,29]. As shown in Figure 3a,c, the Ce^3+^/(Ce^3+^ + Ce^4+^) ratio is 0.272 for L-CeO_2_ and 0.266 for P-CeO_2_. Figure 3b,d shows the O1s spectra, with the main peak detected at 529 eV (denoted as O_α_) being related to cerium’s lattice oxygen, while the weaker peak near 531 eV is associated with the surface absorbed oxygen (O_β_) [30]. The O_β_/(O_α_ + O_β_) ratio can be used to explain the concentration of surface oxygen vacancies, which is higher for L-CeO_2_ than for P-CeO_2,_ and which is consistent with the results for Ce^3+^ content. The results of the XPS tests indicate that L-CeO_2_ has more oxygen vacancies, providing more reactive sites.

Pore information of the CeO_2_ was examined by N_2_ adsorption and desorption experiments, based on which, the pore diameters and pore volumes were determined by the BJH method using the desorption branch of the isotherm, and their surface areas were analyzed by the BET method. As shown in Figure S2, L-CeO_2_ and P-CeO_2_ exhibited type II adsorption/desorption isotherms, and the specific surface areas of both were similar. It is noteworthy that L-CeO_2_ has a wider pore size distribution and more mesopores with a larger pore size (average pore size of 26.85 nm). As shown in Table S2, the BJH pore volume of L-CeO_2_ is as high as 0.087 cm^3^/g, while that of P-CeO_2_ is lower at 0.033 cm^3^/g. Mesoporous cerium oxide particles have more active reaction sites than solid nanoparticles [31,32]. The increased pore volume of L-CeO_2_ suggests that it may allow flammable volatiles to be trapped in the channels, resulting in low heat release and smoke production, while free radicals may also enter the pores and be quenched, thereby slowing down combustion [33].

### 2.2. The Flame-Retardant Test

The flame-retardant properties of the composites were evaluated by measuring the limiting oxygen index (LOI) and conducting UL 94 vertical burning tests. As shown in Table 1, pure PP exhibited a low LOI of 17.8% and no UL 94 rating. When 19 wt.% IFR was incorporated (sample PP-2#), the LOI improved to 29.4%, and the composite achieved a V-2 rating. The incorporation of 1 wt.% of CeO_2_ as a co-flame retardant significantly enhanced the flame retardancy. Specifically, the addition of L-CeO_2_ (PP-3#) resulted in an LOI of 32.6% and a V-0 UL 94 rating, while P-CeO_2_ (PP-4#) increased the LOI to 31.0% with the same V-0 rating (a digital photograph of the sample after UL 94 testing is shown in Figure S3). These findings demonstrate that CeO_2_, particularly in the layered morphology, acts as a highly effective synergistic flame retardant, improving the overall efficiency of the IFR system.

### 2.3. Characterization of Flame-Retardant Composites

Density measurements of the composites were carried out and the densities of PP-2#, PP-3#, and PP-4# were found to be 0.890, 0.880, and 0.893 g/cm^3^, respectively. The incorporation of CeO_2_ had almost no effect on the IFR/PP composites. The SEM images (Figure S4) showed that CeO_2_ with different morphologies were well dispersed in the PP matrix.

### 2.4. Cone Calorimeter Analysis of IFR Composites

**Figure 4:**
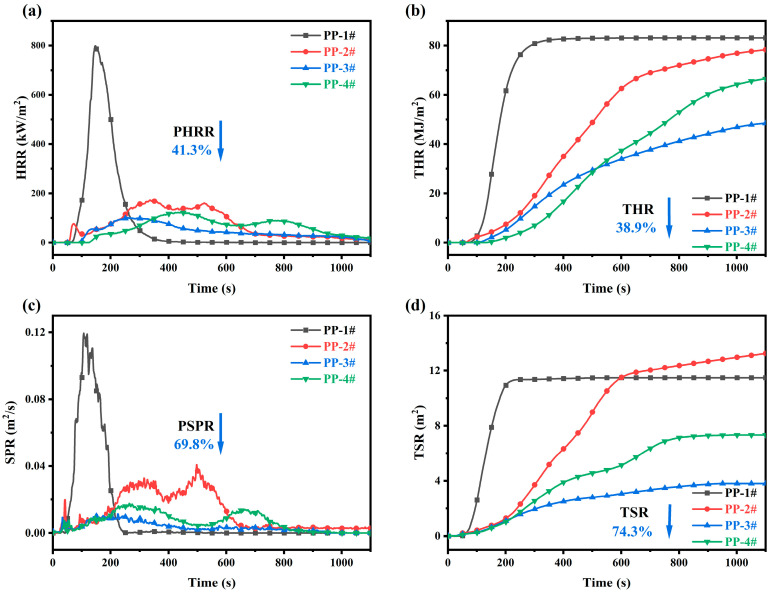
(**a**) HRR, (**b**) THR, (**c**) SPR, and (**d**) TSR curves of PP and PP composites.

Cone calorimetry is widely regarded as one of the most effective methods for evaluating the combustion behavior of polymers under conditions that simulate real fire scenarios [34]. The flammability of the PP composites was assessed through cone calorimetry, with key parameters such as heat release rate (HRR), total heat release (THR), smoke production rate (SPR), and total smoke release (TSR) of PP and PP composites, as presented in Figure 4. The PHRR value of PP-1# was 798.2 kW/m^2^ and the THR value was 83.10 MJ/m^2^, as shown in Figure 4a,b and Table S3. The PHRR and THR of PP-2# decreased after the introduction of IFR, and the PHRR and THR values of the flame-retardant PP composites with added CeO_2_ decreased further. The PHRR value of PP-3# with the addition of L-CeO_2_ was 101.06 kW/m^2^ and the THR value was 48.57 MJ/m^2^. The PHRR and THR values of PP-3# decreased by 41.3% and 38.9%, respectively, compared with that of PP-2#.

In addition to heat release, the addition of CeO_2_ was also found to significantly reduce the smoke generation of the PP composites during combustion. As shown in Figure 4c,d and Table S3, the smoke release rate (SPR) and total smoke production (TSP) of the PP composites exhibited a significant reduction with the addition of CeO_2_ compared to PP-2#. For example, the peak SPR and TSR of PP-3# were reduced by up to 69.8% and 74.3%, respectively, compared to PP-2#. The flame retardancy of PP-3# was significantly improved compared to PP-2#, and these improvements are attributed to the formation of a stable and dense carbonaceous layer during combustion, which serves as an effective physical barrier to heat and mass transfer (as shown in Figure S5).

In addition, the time to PHRR (t-PHRR) of PP-3# is reduced compared to other flame-retardant PP composites, mainly due to its earlier decomposition [35]. This phenomenon is also observed in the ignition times of the composites; e.g., PP-4# has a TTI of 41 s, while PP-3# has a TTI of only 39 s. The fire performance index (FPI), defined as the ratio of time to ignition (TTI) to PHRR, was considerably higher for PP-3# than for the other composites, as shown in Table S3. A higher FPI indicates improved fire safety and a lower fire risk [36]. Thus, PP-3# composites are more fire-safe than other composites.

### 2.5. TGA of IFR Composites

**Figure 5:**
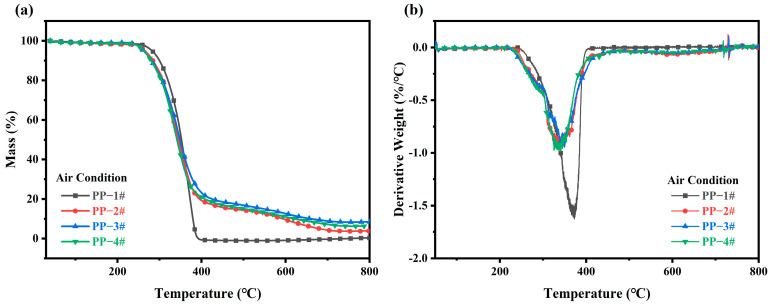
(**a**) TGA and (**b**) DTG curves of PP and PP composites under air atmosphere.

Thermal gravimetric analysis (TGA) and derivative thermogravimetry (DTG) were used to evaluate the thermal stability of the composites under an air atmosphere [37]. As shown in Figure 5 and Table S4, the onset decomposition temperature (T_5%_) of the PP/IFR composites decreased slightly with the addition of CeO_2_, likely due to the catalytic role of CeO_2_ in promoting earlier char formation. However, the final residue at 800 °C (*W*_800_) increased significantly for the CeO_2_-containing composites. L-CeO_2_ enhances thermal stability, elevating the residual char yield to 8.53% at 800 °C in air, a 2.2-fold increase compared to the IFR-only composite (3.87%). This indicates that L-CeO_2_ is more effective in promoting carbonization and enhancing the thermal stability of the PP/IFR composites than P-CeO_2_. As shown in Figure 5b, pure PP exhibits a one-step weight loss process, whereas the weight loss process of PP composites is divided into two stages. The first stage (200–450 °C) is attributed to the decomposition of the composite and the second stage (500–700 °C) is attributed to the degradation of the carbon layer in the presence of heat and oxygen. The peak thermal weight loss rate of polypropylene composites is significantly lower compared to pure PP.

### 2.6. SEM and Raman Analysis of Carbon Layer Morphology

**Figure 6:**
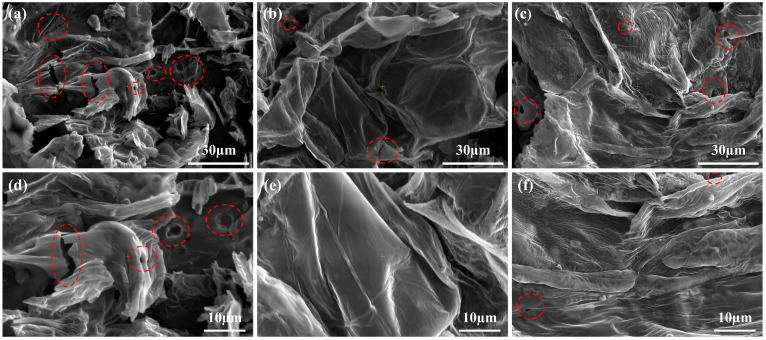
SEM images of char layer after cone calorimetry test: (**a**,**d**) PP-2#, (**b**,**e**) PP-3#, and (**c**,**f**) PP-4#.

**Figure 7:**
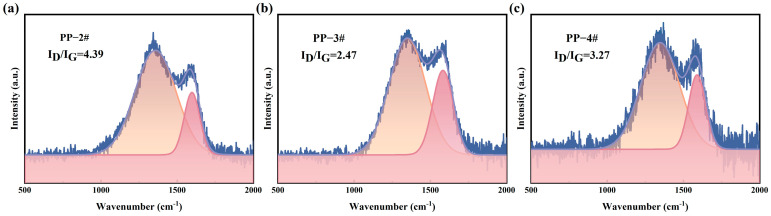
Raman spectroscopy of PP composites: (**a**) PP-2#, (**b**) PP-3#, and (**c**) PP-4#.

The morphology and composition of the char play a vital role in the flame retardancy of polymer composites [38]. SEM images of the char residues (Figure 6) revealed that the char formed by PP-3# was significantly denser and exhibited fewer surface cracks compared to other samples. The structural order of the carbon in char residues can be evaluated using Raman spectroscopy, which identified two characteristic bands associated with carbon materials [39]. The D band, appearing at approximately 1360 cm^−1^, corresponds to disordered carbon structures, while the G band, located around 1600 cm^−1^, is associated with graphitic (ordered) carbon structures. The ratio of the D band to the G band (I_D_/I_G_) is commonly used to estimate the degree of graphitization in carbonaceous materials. Raman spectra (Figure 7) confirmed that the char residue from PP-3# exhibited a lower I_D_/I_G_ ratio, indicating a higher degree of graphitization compared to the other samples. This suggests that the addition of L-CeO_2_ promotes the formation of a more ordered and thermally stable carbon structure. The increased graphitization contributes to enhanced flame retardancy by inhibiting further combustion and reducing heat transfer, as a more graphitic char layer acts as a more effective barrier against heat and mass flow.

### 2.7. FTIR Analysis of IFR Composites

**Figure 8:**
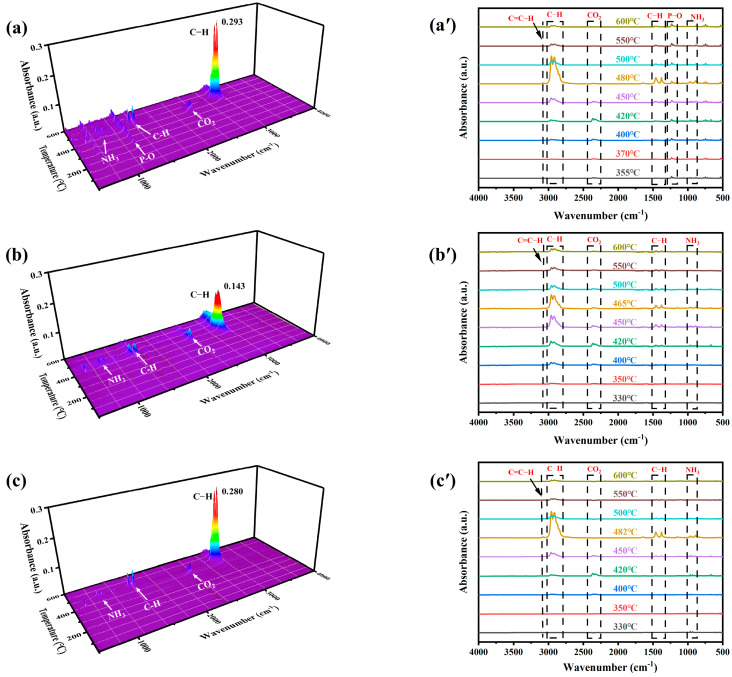
3D FTIR spectra and the corresponding separated FTIR spectra of the degradation products of (**a,a’**) PP-2#, (**b,b’**) PP-3#, and (**c,c’**) PP-4#.

The thermal degradation products of the samples PP-2#, PP-3#, and PP-4# were analyzed using TG-FTIR to identify the released gaseous species during combustion. As depicted in Figure 8, the characteristic absorption peaks of ammonia (NH_3_) were detected at approximately 330 °C in the spectra of both PP-3# and PP-4#, which is earlier than the corresponding peaks observed in PP-2#. This early released NH_3_ facilitated the expansion of the char layer on the composite surface, forming a robust physical barrier that slowed the thermal degradation of the material [40]. Moreover, the absence of P-O absorption bands in the 1050–1250 cm^−1^ range in the spectra of PP-3# and PP-4# suggests a strong interaction between CeO_2_ and the IFR system. This interaction likely contributes to the retention of phosphorus-containing compounds in the char layer as P-O-C crosslinked structures, enhancing flame retardancy [41].

Further analysis of the cumulative FTIR spectra in Figure S6, obtained by integrating the absorption signals across the temperature range, revealed important differences in the emission of small hydrocarbon species among the samples. The released hydrocarbons of PP-4# were approximately 5.4% fewer than those of PP-2#, while PP-3# showed a significant 60% reduction in hydrocarbon emissions. This suggests that the synergistic action of L-CeO_2_ and the IFR system in PP-3# effectively captures small volatile hydrocarbons within the char, promoting their involvement in the carbonization process. The result is a denser, more thermally stable char layer, which plays a critical role in improving flame retardancy by reducing heat transfer and limiting further combustion [38].

These findings demonstrate the enhanced flame-retardant performance of the L-CeO_2_/IFR system, attributed to its ability to accelerate NH_3_ release, retain phosphorus compounds in the char layer, and reduce the emission of combustible hydrocarbons.

### 2.8. Synergistic Flame-Retardant Mechanism

**Figure 9:**
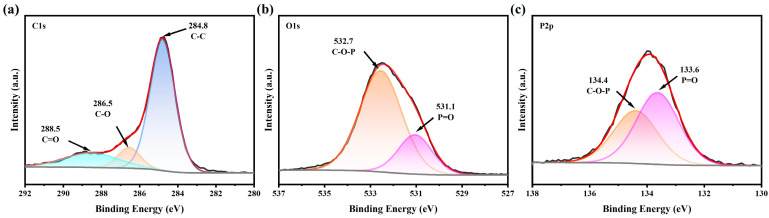
(**a**) C1s, (**b**) O1s, and (**c**) P2p XPS spectra of char residue of PP-3# after the cone calorimeter test.

The residual carbon of PP-3# after cone calorimetry was characterized using X-ray photoelectron spectroscopy (XPS). As shown in Figure 9, the C1s spectrum revealed binding energy peaks at 284.8 eV, 286.5 eV, and 288.5 eV, which are attributed to C-C bonds in aromatic structures or C=C bonds in aliphatic chains, as well as C-O and C=O groups in the P-O-C structures, respectively [35,42,43]. The O1s spectrum showed binding energy peaks at 531.1 eV and 532.7 eV, corresponding to =O in P=O bonds and -O- in P-O-C structures, respectively [44,45]. Additionally, the P2p spectrum exhibited binding energy peaks at 133.6 eV and 134.4 eV, which are associated with P-O-C and P=O bonds, respectively [42,46]. These results suggest that the degradation products, such as phosphoric acid and polyphosphoric acid, generated during the high-temperature decomposition of the L-CeO_2_/IFR system, are retained in the char layer in the form of P=O. Furthermore, they facilitate the formation of extensive P-O-C crosslinked structures during the char-forming process, which significantly enhances the strength and barrier properties of the residual char.

**Figure 10:**
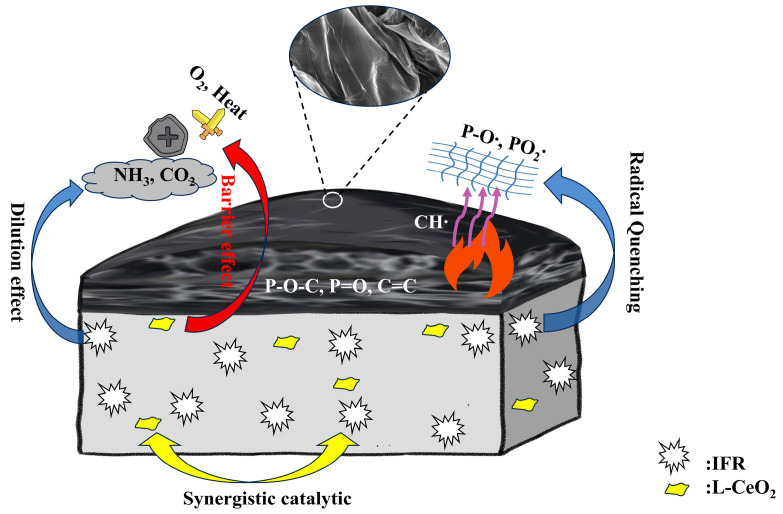
Possible synergistic flame-retardant mechanism of the PP/IFR/L-CeO_2_ system.

Based on these findings, a synergistic flame-retardant mechanism for L-CeO_2_ in the PP/IFR system is proposed (Figure 10). In the condensed phase, L-CeO_2_ catalyzes the formation of a P-O-C crosslinked network within the carbonized layer, improving the mechanical integrity and barrier function of the char. In the gas phase, inert gases such as NH_3_ and CO_2_, released from the IFR system, dilute the oxygen and fuel gases, thereby inhibiting combustion. The enhanced flame retardancy of L-CeO_2_-added polypropylene composites was attributed to their higher specific surface area, larger pore size, and larger pore volume, which facilitated the improvement of catalytic activity and contributed to the improvement of flame retardancy and thermal stability. Meanwhile, the 2D layered structure of L-CeO_2_ further enhances its efficacy as a barrier, limiting the diffusion of heat and oxygen into the underlying material.

### 2.9. Mechanical Properties Analysis of IFR Composites

**Figure 11:**
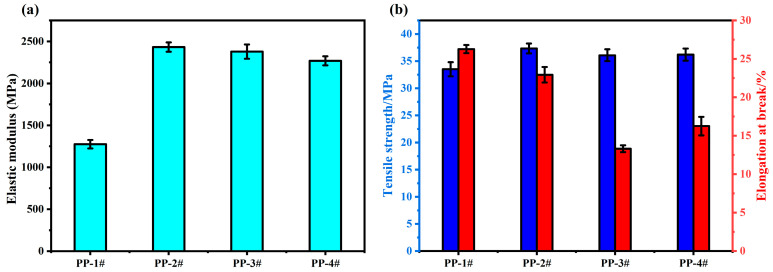
Modulus of elasticity (**a**), and tensile strength and elongation at break (**b**) of PP and PP composites.

The tensile properties of the composites were evaluated, and the results are presented in Figure 11 and Table S5. The addition of the IFR system disrupted the ordered structure of the PP matrix, and the elongation at break (ε) of the PP composites was reduced compared with that of pure PP [47]. The tensile strengths (σ) of the PP composites were slightly improved, by 11.4%, 7.7%, and 8.0% for PP-2#, PP-3#, and PP-4#, respectively, compared with that of pure PP. Unlike elongation at break (ε) and tensile strength (σ), the modulus of elasticity (*E*) of the flame-retardant composites was significantly increased, exhibiting excellent rigidity. The reason for the high rigidity of the flame-retardant composites may be the formation of physical cross-links between the polymer matrix and the flame-retardant particles, which inhibits the rearrangement and orientation of some kinematic units of the polymer matrix during the tensile process [48].

**Figure 12:**
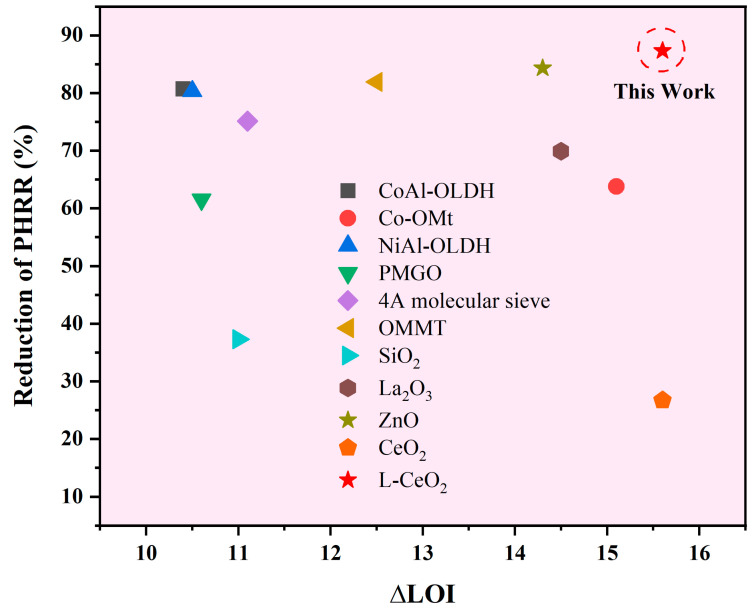
Comparison of PHRR and LOI variation of flame-retardant PP composites.

In order to visually evaluate the flame retardancy of the PP/IFR/L-CeO_2_ composite, Figure 12 shows the difference in flame retardancy between it and previously reported flame-retardant PP composites achieving UL 94 V-0 ratings. Detailed data and relevant references are listed in Table 2. Among all the reported flame-retardant PP systems, the PP/IFR/L-CeO_2_ system prepared in this work shows a relatively large decrease in both PHRR and THR and a significant increase in LOI value, which demonstrates excellent flame-retardant performance.

## 3. Materials and Methods

### 3.1. Materials

Polypropylene (PP) in pellet form (model: T30S) was sourced from Sinopec Zhenhai Refining & Chemical Company (Ningbo, China). Piperazine pyrophosphate (PAPP, model: XS-PPAP-100) was supplied by Zhejiang Xusen Non-halogen Smoke Suppressing Fire Retardants Co., Ltd. (Jiaxing, China), while melamine polyphosphate (MPP, model: 2021122701) was provided by Hefei Wanran New Material Technology Co., Ltd (Hefei, China). P-CeO_2_ (model: 20220906) was obtained from Inner Mongolia Zhongke Lanthanum Cerium Rare Material Technology Co., Ltd (Baotou, China). NH_4_HCO_3_, cerium chloride heptahydrate CeCl_3_∙7H_2_O, model: C211821000), and polyvinyl alcohol (PVA molecular weight range 10,000, model: 30153160) were purchased from Sinopharm Chemical Reagent Co., Ltd (Shanghai, China). All materials were used as received without further purification.

### 3.2. Synthesis of L-CeO_2_

L-CeO_2_ was synthesized using a controlled precipitation method. Initially, 12 g of polyvinyl alcohol (PVA) was dissolved in 500 mL of hot deionized water (solution 1). In parallel, 40 g of CeCl_3_·7H_2_O was dissolved in 500 mL of deionized water, and the pH was adjusted to 5.0 (solution 2). Solution 1 was slowly introduced into solution 2 under continuous stirring at room temperature, forming solution 3. Separately, 4 mol of ammonium bicarbonate was dissolved in 1 L of deionized water (solution 4) and added dropwise into solution 3 with constant stirring. A white precipitate was formed, which was then filtered, washed thoroughly with deionized water, and dried at 80 °C. The dried powder was subjected to calcination in a muffle furnace at 550 °C for 4 h to obtain L-CeO_2_.

### 3.3. Preparation of Polypropylene Composites

The composites were prepared by melt blending PP (81 wt.%), IFR (18 wt.% PAPP:MPP = 2:1), and 1 wt.% CeO_2_ (L-CeO_2_ or P-CeO_2_) using a torque rheometer (190 °C, 30 rpm, 8 min). Following melt blending, the composites were transferred into preheated molds and processed by hot pressing. The molding process involved preheating (4 min), hot pressing (190 °C, 5 Mpa, 3 min), and cooling (3 min). The resultant materials were formed into sheets of varying dimensions, depending on the intended tests.

### 3.4. Characterization

The structural properties of P-CeO_2_ and L-CeO_2_ were characterized using X-ray diffraction (XRD) on a Miniflex 600 diffractometer (Rigaku Corporation, Tokyo, Japan) with Cu Kα radiation. The scans were conducted over a range of 5–80° at a scanning rate of 10°/min.

The microstructure of the prepared composites was analyzed via scanning electron microscopy (SEM) using a Apreo S microscope (Thermo Fisher, Prague, Czech Republic). Energy dispersive spectroscopy (EDS, XFlash 6-60, Bruker, München, Germany) was performed on PP composite cross sections.

The particle size distribution and zeta potential of the cerium oxide were measured using a multi-angle particle size high-sensitivity zeta potential analyzer (DLS & Zeta PALS, Omni, Brookhaven, NY, USA).

The specific surface area, pore volume, and pore size distribution of the cerium oxide were evaluated using a specific surface area analyzer (BET, Quantachrome Autosorb-iQ, Contamination Instruments, San Francisco, CA, USA).

The thermal stability of the composites was assessed using a synchronous thermal analyzer (TGA/DSC1, Mettler-Toledo, Zurich, Switzerland). Samples were heated from 30 °C to 800 °C at a rate of 10 °C/min under an air atmosphere.

The limiting oxygen index (LOI) was measured using an oxygen index instrument (OI, Mortis Combustion Technology, Kunshan, China) in accordance with ISO 4589-2:1996 [58], with sample dimensions of 125 mm in length, 6.5 mm in width, and 3 mm in thickness.

Vertical flame retardancy was evaluated using the UL 94 test in a horizontal-vertical burning tester (TTech-GBT2408, TESSTECH, Suzhou, China) following ASTM D3801-2020a [59]. Sample dimensions for the UL 94 test were 125 mm in length, 10 mm in width, and 3 mm in thickness, and each test was performed in quintuplicate to ensure reproducibility.

The combustion properties of the composites were examined using a cone calorimeter (CCT, Mortis Combustion Technology, China) in accordance with ISO 5660-1:2015 [60]. The samples with dimensions of 100 mm in length, 100 mm in width, and 3 mm in thickness were exposed to a heat flux of 35 kW/m².

The mechanical properties were measured using a universal tensile testing machine (Instron 2365, Instron corporation, Boston, MA, USA) based on ISO 527-1-1993 [61] standards at a crosshead speed of 50 mm/min. Dumbbell-shaped specimens were used for testing, with dimensions of 160 mm in length, 10 mm in width, and 4 mm in thickness.

Raman spectra were obtained using a confocal Raman spectrometer (BWS465-785S, B&W Tek, Shanghai, China) with an excitation wavelength of 785 nm.

Oxygen vacancies in cerium oxide and residual carbon content in the PP composites were analyzed using X-ray photoelectron spectroscopy (XPS, Thermo Scientific K-Alpha, Thermo Fisher, Waltham, MA, USA) with Al Kα radiation (hv = 1486.6 eV).

Thermogravimetric analysis (TGA) coupled with Fourier-transform infrared (FTIR) spectroscopy(TG-IR) was performed using a STA-2500-iS50 spectrometry (Netzsch, Selb, Germany; Thermo Fisher, Waltham, MA, USA) with a system flow rate of 50 mL/min. The temperature range for the analysis was set from 30 °C to 600 °C, and the FTIR spectra were recorded over the wavelength range of 500 to 4000 cm^−1^.

## 4. Conclusions

This study systematically investigated the synergistic effects of CeO_2_ with different morphologies on the flame retardancy, thermal stability, and mechanical properties of intumescent flame-retardant polypropylene (PP/IFR) composites. The results demonstrated that the incorporation of L-CeO_2_ significantly improved the flame-retardant efficiency of the IFR system, as indicated by an increased limiting oxygen index (LOI), a reduced heat release rate (HRR), and a lower total smoke production (TSP). L-CeO_2_ outperformed P-CeO_2_, which is attributed to its larger surface area and superior catalytic activity, promoting the formation of a stable and dense char layer during combustion. Thermal analysis further showed that L-CeO_2_ enhanced the carbonization process, resulting in higher residual char and improved thermal stability at elevated temperatures. Additionally, the enhanced mechanical properties, including increased tensile strength and rigidity, suggest that L-CeO_2_ strengthens the composite structure by improving the interaction between the flame retardants and the PP matrix. The improved tensile strength and modulus of elasticity make the composites promising for applications in scenarios requiring high stiffness, such as the automotive industry and construction. Overall, this study highlights the potential of CeO_2_, particularly in its layered morphology, as an effective synergistic flame retardant in PP/IFR composites, offering valuable insights for developing advanced flame-retardant materials with improved fire resistance and mechanical integrity. These findings pave the way for further research into the mechanisms behind the synergistic effect of CeO_2_ and its applications in other polymer systems, contributing to more efficient and environmentally friendly flame-retardant solutions.

## Figures and Tables

**Table 1:** LOI and UL 94 data of the samples.

Sample	Components (wt.%)				UL 94	t_1_/t_2_ (s) ^b^	Dripping	Cotton Ignited	LOI (%)
	PP	IFR ^a^	L-CeO_2_	P-CeO_2_					
PP-1#	100				NR	>60	Y	Y	17.8
PP-2#	81	19			V-2	1.3/18.5	Y	Y	29.4
PP-3#	81	18	1		V-0	0.5/7.2	N	N	32.6
PP-4#	81	18		1	V-0	0.5/5.5	N	N	31.0

^a^ IFR consists of PAPP and MPP with a mass ratio of 2:1. ^b^ t_1_/t_2_: flame duration after the first and second ignition.

**Table 2:** Comprehensive performance comparison of flame-retardant PP composites (UL94 V-0 rated).

Synergistic Flame Retardant	Content (wt.%)	Variation of THR (%)	Variation of PHRR (%)	∆LOI	Refs.
CoAl-OLDH	3	36.9	80.7	10.4	[49]
Co-OMt	4	17.6	63.8	15.1	[50]
NiAl-OLDH	5	19.1	80.3	10.5	[51]
PMGO	5	40.2	61.5	10.6	[52]
4A molecular sieve	1	17.1	75.1	11.1	[53]
OMMT	3	17.8	81.9	12.5	[54]
SiO_2_	1	9.1	37.3	11.0	[55]
La_2_O_3_	1	21.1	69.9	14.5	[56]
ZnO	0.5	28.1	84.3	14.3	[57]
CeO_2_	1	16.5	26.7	15.6	[20]
L-CeO_2_	1	41.6	87.3	14.8	This Work

## Data Availability

Data are contained within the article and Supplementary Materials.

## Supplementary Materials

The following supporting information can be downloaded at: https://www.mdpi.com/article/10.3390/molecules30102102/s1, Figure S1: X-ray diffraction patterns of different morphologies of CeO2. Figure S2: BET data of CeO2 with different morphologies: (a) Isothermal adsorption and desorption curves of N2, (b) Pore size distribution. Figure S3: Digital photographs of samples after UL 94 testing. Figure S4: SEM images of cross sections of PP composites, (a) PP-3#; (b) PP-4#. Figure S5: Digital photos of char layer after cone calorimetry test. Figure S6: Cumulative FTIR spectra of the degradation products of PP composite; Table S1: Zeta Potential data of CeO2. Table S2: BET data of CeO2. Table S3: Cone calorimetry test data of PP and PP composites. Table S4: TGA data of PP composites. Table S5: Tensile test results of flame retardant composites.
