# Supplementary material for: Morphology-Engineered CeO2 as a Synergistic Flame Retardant in Polypropylene/Intumescent Systems: Mechanisms and Performance Enhancement
Source: Molecules. 2025 May 9;30(10):2102. doi: 10.3390/molecules30102102 (PMC12113799; doi:10.3390/molecules30102102)
Supplement: Supplementary file 1 [file molecules-30-02102-s001.zip › molecules-3596832-supplementary.pdf]

Supplementary material

Morphology-Dependent Synergistic Flame Retardancy of CeO_2 in Polypropylene with Intumescent Flame Retardants

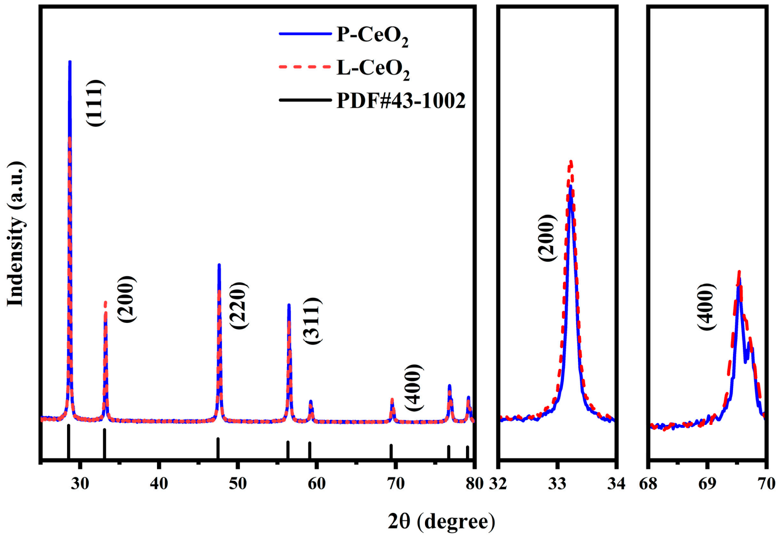

Supplementary Figure S1: X-ray diffraction patterns of different morphologies of CeO_2.

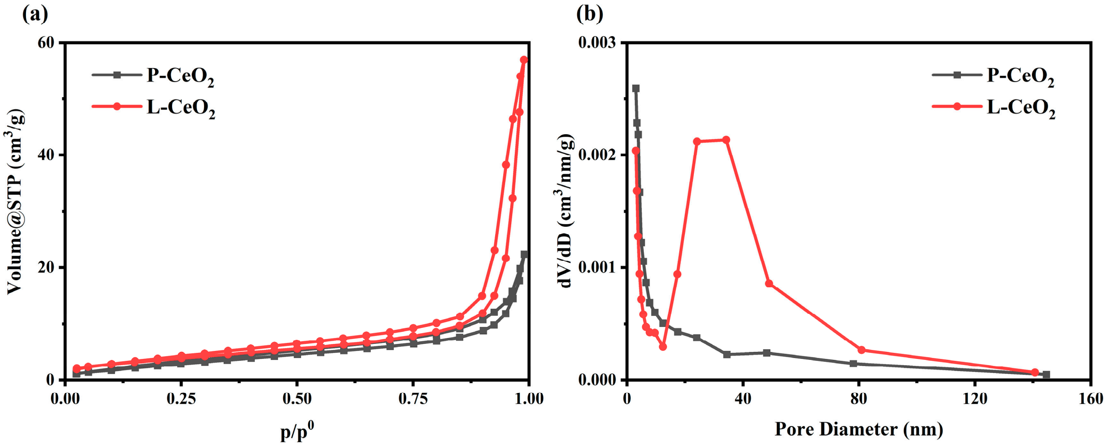

Supplementary Figure S2: BET data of CeO_2 with different morphologies: (a) Isothermal adsorption and desorption curves of N_2, (b) Pore size distribution.

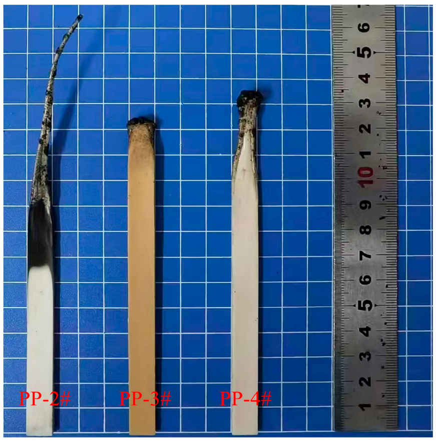

Supplementary Figure S3: Digital photographs of samples after UL 94 testing.

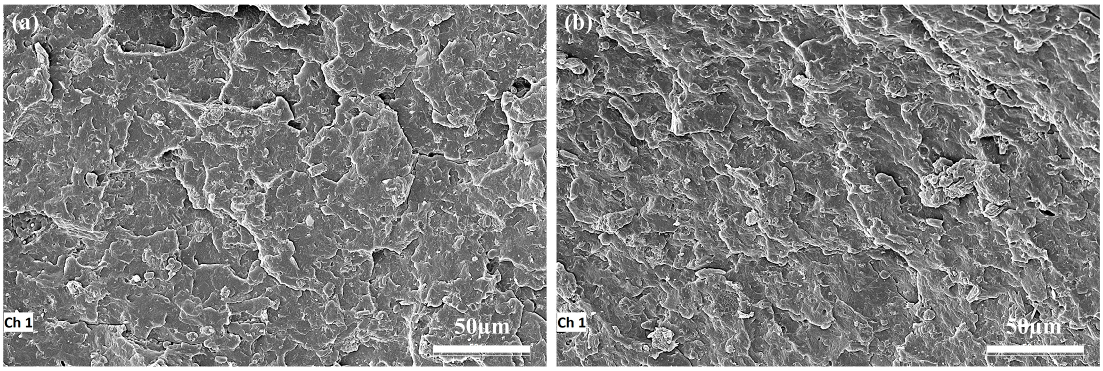

Supplementary Figure S4: SEM images of cross sections of PP composites，

(a) PP-3#; (b) PP-4#

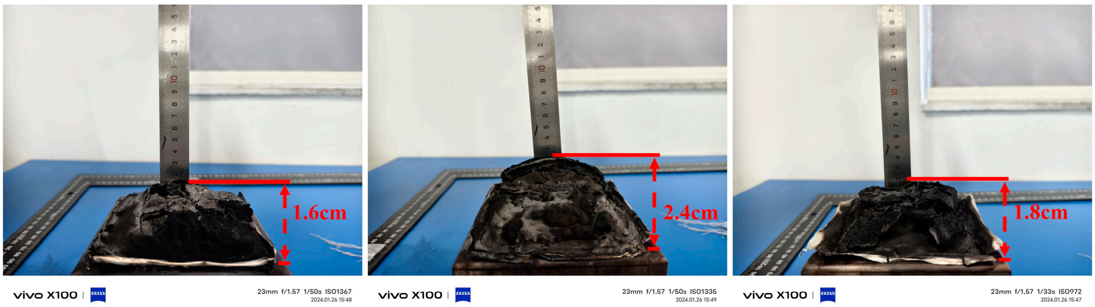

PP-2# PP-3# PP-4#

Supplementary Figure S5: Digital photos of char layer after cone calorimetry test.

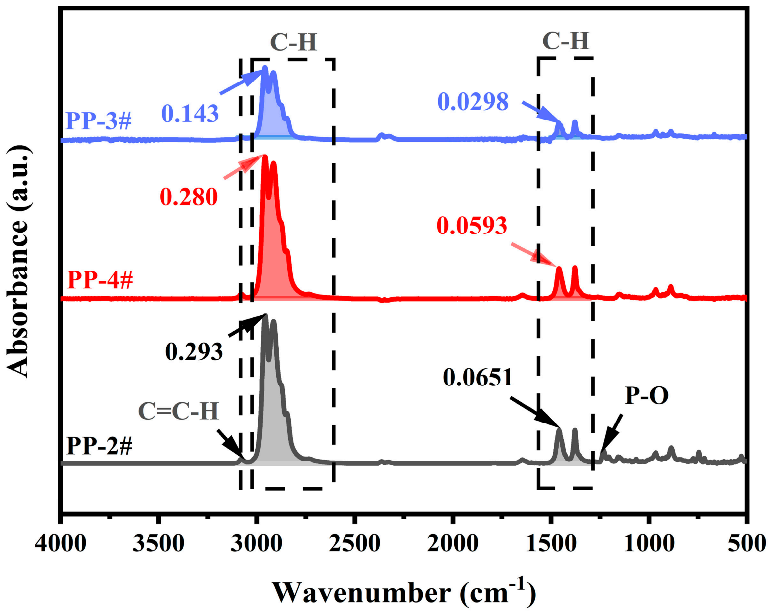

Supplementary Figure S6: Cumulative FTIR spectra of the degradation products of PP composite.

Supplementary Table S1: Zeta Potential data of CeO_2.

	L-CeO_2	P-CeO_2
	36.31	-19.79
Zeta Potential (mV)	39.94	-19.43
	34.13	-15.11
Mean	36.78	-18.11

Supplementary Table S2: BET data of CeO_2.

Sample	BET Surface area m^2/g	Average pore Diameter (nm)	BJH Pore Volume (cm^3/g)
P-CeO_2	11.61	11.92	0.033
L-CeO_2	13.11	26.85	0.087

Supplementary Table S3: Cone calorimetry test data of PP and PP composites.

**Classification**	**PP-1#**	**PP-2#**	**PP-3#**	**PP-4#**
TTI (s)	27	33	39	41
PHRR (kW/m^2)	798.2	172.24	101.06	122.46
THR (MJ/m^2)	83.10	79.45	48.57	67.64
t-PHRR (s)	148	335	259	445
TSR (m^2)	11.48	14.81	3.81	7.33
PSRR (m^2/s)	0.1195	0.0408	0.0123	0.0176
RC (*wt.*%)	6.4	15.7	40.9	24.6
FPI	0.034	0.195	0.386	0.335

TTI: Time to ignition

PHRR: Peak heat release rate, HRR: The heat release rate, THR: Total heat release

SPR: Smoke production rate, PSPR: The peak of smoke release rate, TSR: Total smoke release

RC: The residue char

FPI: Fire performance index (FPI=TTI/PHRR)

Supplementary Table S4: TGA data of PP composites.

Sample	$T_{5\%}$ (°C)	T_{max} (°C)	W_{800} (%)
PP-1#	282	372	0.38
PP-2#	266	335	3.87
PP-3#	262	349	8.53
PP-4#	263	339	6.36

Supplementary Table S5: Tensile test results of flame retardant composites.

Sample	Tensile strength/MPa	Elongation at break/%	Elastic modulus/MPa
PP-1#	33.51±1.32	26.28±0.53	1274.4±50.15
PP-2#	37.34±0.92	22.93±1.01	2432.18±55.27
PP-3#	36.09±1.09	13.30±0.45	2378.43±84.52
PP-4#	36.20±1.10	16.25±1.20	2268.86±54.50
